# Effects of Long-Term Walnut Supplementation on Body Weight in Free-Living Elderly: Results of a Randomized Controlled Trial

**DOI:** 10.3390/nu10091317

**Published:** 2018-09-18

**Authors:** Edward Bitok, Sujatha Rajaram, Karen Jaceldo-Siegl, Keiji Oda, Aleix Sala-Vila, Mercè Serra-Mir, Emilio Ros, Joan Sabaté

**Affiliations:** 1 Center for Nutrition, Healthy Lifestyle and Disease Prevention, School of Public Health, Loma Linda University, Loma Linda, CA 92350, USA; srajaram@llu.edu (S.R.); kjaceldo@llu.edu (K.J.-S.); koda@llu.edu (K.O.); jsabate@llu.edu (J.S.); 2 Department of Nutrition & Dietetics, School of Allied Health Professions, Loma Linda University, Loma Linda, CA 92350, USA; 3 Lipid Clinic, Endocrinology and Nutrition Service, Institut d’Investigacions Biomèdiques August Pi Sunyer (IDIBAPS), Hospital Clínic, Barcelona 08036, Spain; asala@clinic.cat (A.S.-V.); SERRAMIR@clinic.cat (M.S.-M.); EROS@clinic.cat (E.R.); 4 Ciber Fisiopatología de la Obesidad y Nutrición (CIBEROBN), Instituto de Salud Carlos III (ISCIII), Madrid 28029, Spain

**Keywords:** nuts, walnuts, body weight, adiposity, obesity, elderly, energy

## Abstract

*Objective*: To assess the effects of chronic walnut consumption on body weight and adiposity in elderly individuals. *Methods*: The Walnuts and Healthy Aging study is a dual-center (Barcelona, Spain and Loma Linda University (LLU)), 2-year randomized parallel trial. This report concerns only the LLU cohort. Healthy elders (mean age 69 year, 67% women) were randomly assigned to walnut (*n* = 183) or control diets (*n* = 173). Subjects in the walnut group received packaged walnuts (28–56 g/day), equivalent to ≈15% of daily energy requirements, to incorporate into their habitual diet, while those in the control group abstained from walnuts. Adiposity was measured periodically, and data were adjusted for in-trial changes in self-reported physical activity. *Results*: After 2 years, body weight significantly decreased (*p =* 0.031), while body fat significantly increased (*p =* 0.0001). However, no significant differences were observed between the control and walnut groups regarding body weight (−0.6 kg and −0.4 kg, respectively, *p =* 0.67) or body fat (+0.9% and +1.3%, respectively, *p =* 0.53). Lean body mass, waist circumference, and waist-to-hip ratio remained essentially unchanged. Sensitivity analyses were consistent with the findings of primary analysis. *Conclusion*: Our findings indicate that walnuts can be incorporated into the daily diet of healthy elders without concern for adverse effects on body weight or body composition.

## 1. Introduction

Obesity in older adults continues to be a major public health challenge in the United States (U.S.) and around the world [1,2]. More than a third of U.S. adults aged 60 years and over are considered as being obese [1,2], a trend that will continue to rise in parallel with the pace of population aging [3]. Excess body fat is an important risk factor for morbidity and mortality from heart disease, diabetes mellitus, dyslipidemia, and metabolic syndrome [4]. In older adults, obesity imposes further functional limitations on top of declining physical function and adversely affects quality of life [5].

Over the years, mounting scientific evidence has shown that consuming nuts in moderate amounts is associated with reduced risk of coronary heart disease [6]. Nuts have a high total fat content (mostly as mono- and polyunsaturated fat), ranging from 46% in cashews and pistachios to 76% in macadamia nuts, and provide 20–30 kJ per gram [7]. They are also rich in protein, fiber, vitamins, minerals, phytosterols, and polyphenols [8]. Following the approval of a qualified health claim by the Food and Drug Association (FDA) supporting the inclusion of 1.5 ounces (42 g) walnuts in the daily diet [9], several agencies including the American Heart Association (AHA) and the Academy of Nutrition and Dietetics (AND) recommend the inclusion of nuts in the daily diet to further heart health [10,11]. Notwithstanding the recommendations, there is a common perception that consuming nuts on a regular basis may lead to unwanted increase in body weight and a higher risk of developing overweight or obesity. However, a meta-analysis of 33 clinical trials assessing the effects of nut-enriched diets compared with various control diets on changes in body weight, body mass index (BMI), and waist circumference indicates that nut-enriched diets do not increase adiposity [12]. In fact, including nuts as part of a weight loss regimen can lead to greater weight loss than simply following a low-fat diet [13]. It is worth noting that many of the trials included in the meta-analysis were conducted over a relatively short period of time (≤6 m) and with mostly young and middle-age adults. Thus, whether long-term inclusion of nuts in the daily, self-selected, unrestricted-calorie diets of elderly subjects results in weight gain remains unclear. 

We had a unique opportunity to clarify this issue within the framework of a 2-year trial testing the effects of walnuts on age-related cognitive decline and macular degeneration in healthy elderly subjects. We were primarily interested in determining if daily consumption of walnuts for an extended period of time induced weight gain in free-living elderly subjects when compared to a similar concurrent group of individuals with low nut consumption.

## 2. Materials and Methods

### 2.1. Study Design and Population

Details of the Walnuts and Healthy Aging (WAHA) study have been published [14]. In brief, it was a randomized dual-center trial, conducted at Loma Linda University (California) and Hospital Clínic (Barcelona, Spain). This opportunistic sub-study concerns data from participants recruited at the Loma Linda site between October 2012 and May 2014. Recruitment for the WAHA study was multi-pronged, and included direct mailings, brochures, flyers, web, and newspaper advertisements. Candidates were pre-screened and excluded from participation if they had morbid obesity, uncontrolled diabetes or hypertension, impaired cognitive function, or bilateral eye conditions preventing visualization of the retina. The present study was conducted according to guidelines laid down in the Declaration of Helsinki. The Institutional Review Board at Loma Linda University approved the study protocol. Written informed consent was obtained from each participant prior to enrollment into the study. 

### 2.2. Intervention

With a parallel design, candidates who met eligibility criteria were randomly assigned to either a walnut (experimental) or control group using a web-based, computerized random number table with stratification by sex and age. Couples entering the study were treated as one number and were randomized into the same group to facilitate compliance. We then utilized the World Health Organization (WHO) formula for energy needs for adults >60 years [15] to estimate individual energy requirements, following which participants received 28, 42, or 56 g (1, 1.5, or 2.0 oz.) of packaged walnuts per day providing ≈15% of their estimated daily energy needs. No advice on food replacement was given and no recipes were provided. Participants in the control group simply continued their habitual diet with no supplementation and with instructions to refrain from eating walnuts or excessive intake of other nuts (>2 servings/week). Simply being in a research study can cause individuals to alter their lifestyle or behavior due to the awareness that they are being watched. This observation is particularly common among studies that collect body measurements. Consequently, we asked participants not to alter their usual lifestyle habits, including physical activity level, while in the study. Participants were largely unaware that adiposity measurements were outcomes of interest in the study. 

### 2.3. Assessment of Diet

We collected 1490 unannounced 24-h telephone diet recalls from study participants during the 2-year period (752 in walnut group and 738 in control group). The diet recalls were obtained at regular intervals to capture variability and seasonality in food intake. Dietary intake data were collected by trained research dietitians and nutrient data obtained using the Nutrition Data System for Research (NDSR) software version 2013 developed by the Nutrition Coordinating Center (NCC), University of Minnesota, Minneapolis, MN [16]. Portion sizes were estimated using common household items; for example, a fist for one baked potato, a deck of cards for a 3-oz serving of meat, or two handfuls for 1-oz of chips or pretzels, as previously described [17]. The dietary recalls were used to determine if subjects in the walnut group consumed their allotted amounts of walnuts and if their counterparts in the control group refrained from deliberate consumption of walnuts. For the walnut group, consumption of walnuts 6–7 days/week (85–100%) was considered excellent compliance and 4–5 days/week (57–71%), as good compliance. Those who consumed walnuts ≤3 days/week were classified as non-compliant. In the control group, participants were considered fully compliant if they refrained from eating walnuts in any of the recalls, or if they consumed no more than 15 g of walnuts on any given day. We also used the red blood cell (RBC) proportion of alpha-linolenic acid (ALA), a nutrient enriched in walnuts, as an objective biomarker [18] to assess adherence to the intervention.

### 2.4. Anthropometry, Body Composition, and Physical Activity

We measured participants’ body weight to the nearest 0.1 kg at baseline and bimonthly. Body fat and lean body mass were measured at baseline, 1-year, and end of study. Body measurements were obtained without shoes or heavy clothing using Tanita^®^ TBF 300A Bioelectrical Impedance Analysis scale (Tanita Corporation of America, Arlington Heights, IL, USA). Participants were asked to avoid exercise or heavy hydration prior to visiting the clinic for body measurements. Height was measured to the nearest 0.1 cm using a wall-mounted stadiometer (Holtain Ltd., Crymych, Dyfed, UK). Waist circumference was measured to the nearest 0.1 cm an inch (2.54 cm) above the umbilicus using a tape measure. Hip circumference was measured to the nearest 0.1 cm at the outermost part of the greater trochanters. The waist-to-hip ratio (WtHR) was computed as the ratio of these circumferences. All measurements were obtained following the Centers for Disease Control (CDC) guidelines for the National Health and Nutrition Examination Survey (NHANES III) anthropometric measurements [19]. We also asked participants to fill in a validated short version of the Minnesota physical activity questionnaire for adult populations [19] at baseline, 1-year, and end of the study. We then applied CDC and American College of Sports Medicine guidelines [20] to compute metabolic equivalent (MET)-hours per week and to categorize general physical activities according to level of intensity (low/sedentary, moderate, and vigorous).

### 2.5. Biomarker Analyses

Detailed procedures for blood collection and analyses in the WAHA study are published [14]. Fasting blood samples were obtained from all participants at baseline and end of the study. To reduce assay variability, all samples were stored and run together in the same laboratory at the end of the study. The RBC proportion of ALA was assessed as described [21] in a random subset of participants (*n* = 105, 51 in the control group and 54 in the walnut group). In brief, cells contained in a 100-μL aliquot of EDTA-collected blood were hemolyzed and spun. The pellet (>99% RBC membranes) was dried, dissolved in 1 mL BF_3_ methanol solution and heated to hydrolyze and methylate glycerophospholipid fatty acids. The fatty acid methyl esters were isolated by adding n-hexane and were separated by gas chromatography using an Agilent HP 7890 Gas Chromatograph equipped with a 30 m × 0.25 μm × 0.25 mm SupraWAX-280 capillary column (Teknokroma, Barcelona, Spain), an autosampler, and a flame ionization detector. The amount of ALA was expressed as a percentage of total identified fatty acids in the RBC sample.

### 2.6. Statistical Analyses

Per protocol analysis was utilized to estimate changes in body measurements. To reduce intra-individual variation, measurements taken in duplicate were averaged and analyses performed on the average. Descriptive statistics are reported as proportions (%) or means ± standard deviations. When appropriate, the ANOVA or chi-square tests were used to assess whether the completers were comparable to non-completers in terms of age, sex, ethnicity, and baseline BMI. Baseline imbalances in demographic, anthropometric, and lifestyle variables between treatment groups were assessed by Chi-square test for independence, two-sample *t*-test, and Fisher’s exact test, as appropriate. The independent samples *t*-test was used to test between group difference in energy and nutrient intake. Changes in body weight and adiposity measures were estimated using linear mixed models with random intercepts and random slopes. Analyses were performed adjusting for in-trial changes in physical activity. The main outcome was change in body weight from baseline to 2 years, with five repeated measurements obtained in between. We also examined changes in body fat, waist circumference, lean body mass, and WtHR at 1-year and end of the study. The predictors for the model were time (as a continuous variable) and intervention (walnut or control group). In the models we included interaction terms for time and intervention (group) by time effects. Three-way interactions between time, intervention, and either age (≤70 years vs. ≥71 years), sex, or ethnicity (white vs. non-white) were also assessed. Changes in ALA as proportion of total identified fatty acids were determined by use of ANOVA, and the relationship between changes in self-reported walnut intake and changes in RBC ALA assessed using Pearson correlation. Assuming a standard deviation of 4 kg, the sample size of 356 participants provided >95% power (with *p* = 0.05) to detect a mean difference of 1 kg between groups. All analyses were performed using Statistical Analysis System (SAS Version 9.4).

## 3. Results

### 3.1. Participants

**Figure 1:**
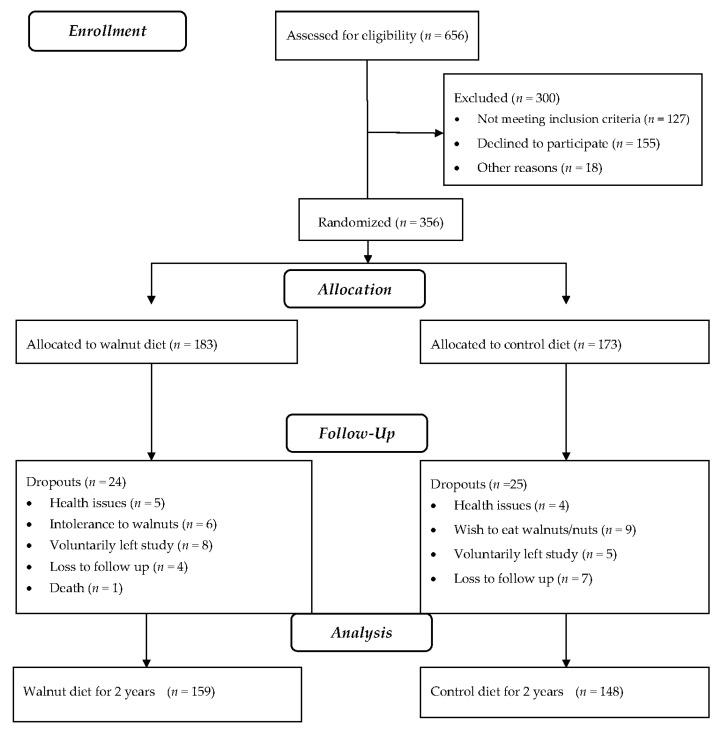
Study flowchart.

Baseline characteristics of 356 subjects who began the study are detailed in Table 1. Overall, the walnut supplement was well accepted and well tolerated by study participants. Forty-nine participants (24 in the walnut group and 25 in the control group) dropped out due to health-related concerns, intolerance to walnuts, loss to follow-up, or undisclosed personal reasons. One death due to esophageal cancer early in the study (unrelated to treatment) occurred in the walnut group. The dropouts did not differ significantly from completers regarding age, sex, ethnicity, or baseline BMI (data not shown). Nine incident cases of constipation and eight of diarrhea were reported in the walnut group during the 2-year study period. Figure 1 is the study flowchart. Data presented are for participants who completed the study (159 from the walnut group and 148 from the control group). 

### 3.2. Compliance with Treatment

Only 1% of dietary recalls from the control group showed intake of trivial amounts of walnuts (<15 g), mostly as an ingredient in recipes and commercially prepared foods such as walnut bread, cookies, or salads. We therefore considered the subjects in the control group to have been 100% compliant with instructions not to consume walnuts. Similarly, 99% of dietary recalls in the walnut group reported consumption of the prescribed amount of walnuts (between 28 and 56 g/day, average 43 g/day). Table 2 shows data of macronutrients based on self-reported intake at two years. On average, energy, total polyunsaturated fatty acids, protein and fiber intake was significantly higher in the walnut group compared to control. The walnut supplement contributed approximately 15% of estimated daily energy needs.

Analysis of baseline RBC fatty acids in a random sub-set of 105 study participants showed similar baseline levels of ALA (mean, 0.30% for the walnut group and 0.28% for the control group; *p* = 0.830). By the end of the study, the mean RBC ALA had increased by 33% in the walnut group and by 14% in the control group (*p* < 0.001). The correlation between 2-year changes in self-reported walnut intake and changes in RBC ALA was significant (*r* = 0.49, *p* < 0.001). 

### 3.3. Changes in Physical Activity and Anthropometric Measurements

**Figure 2:**
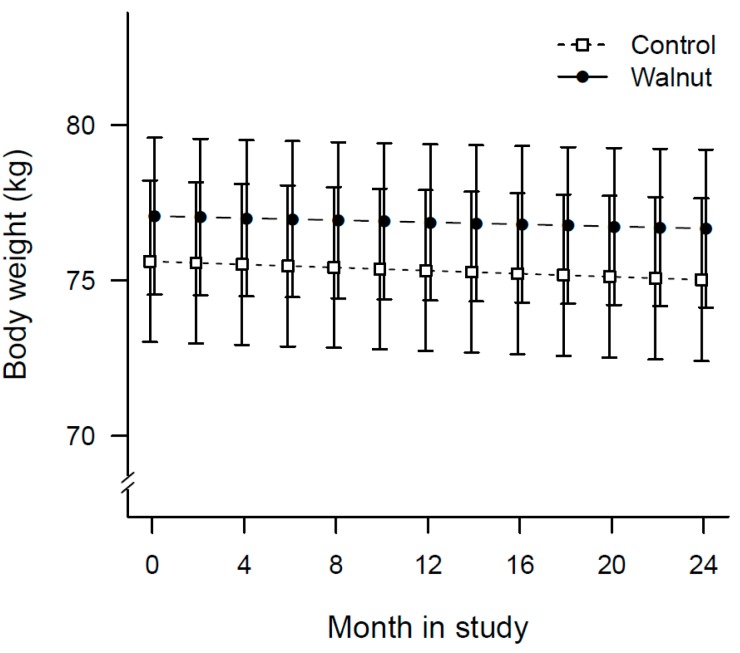
Plot of changes in mean body weight of participants over time by treatment allocation.

Table 3 shows the results of anthropometric measurements. Overall, body weight decreased significantly over time in all study participants (*p* = 0.031). Figure 2 is a plot of the average body weight of participants obtained periodically during clinic visits. Participants in the walnut group lost an average of 0.4 kg compared to 0.6 kg in the control group, with no between group differences (*p* = 0.671). 

**Figure 3:**
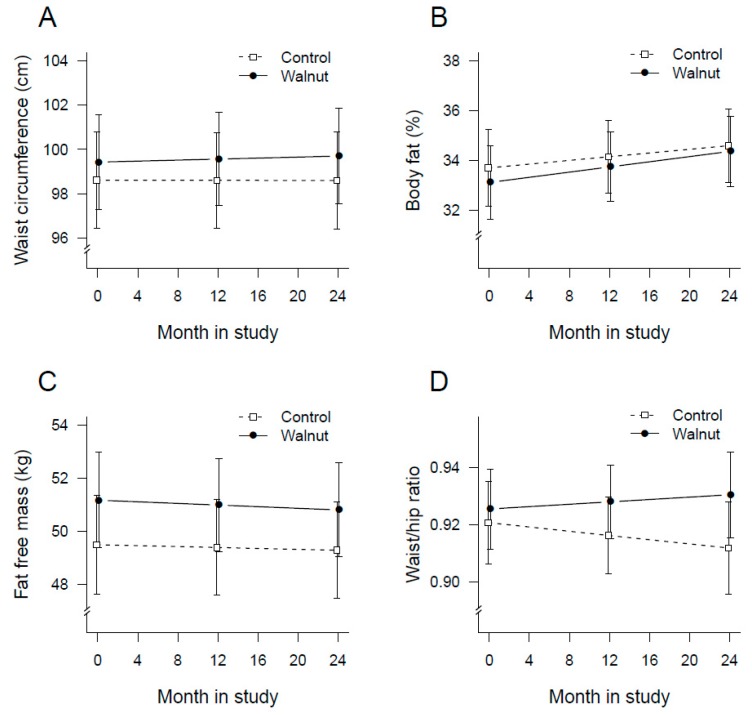
Plot of changes in mean waist circumference (**A**), body fat (**B**), lean body mass (**C**), and waist-to-hip ratio (**D**) over time by intervention group.

Figure 3A is a plot of the average waist circumference of participants at baseline, 1-year, and end of the study. The increase in waist circumference over time was not significant (*p* = 0.680) and there were no between group difference (*p* = 0.651). Figure 3B is a plot of the average body fat at baseline, 1-year, and end of the study. Mean body fat increased significantly in both groups (*p* < 0.001). Participants in the walnut group gained ≈0.9 kg (1.8%) body fat compared to 0.5 kg (0.9%) in the control group (*p* = 0.528 for between group differences). 

Lean body mass decreased by 0.4 kg (0.8%) in the walnut group and by 0.2 kg (0.4%) in the control group. The change in lean body mass over time was not significant (*p* = 0.220) and did not differ between the two groups (*p* = 0.740) (Figure 3C). The change in WtHR over time (Figure 3D) was negligible, −0.009 in the control and +0.005 in the walnut group. Self-reported physical activity increased significantly over time in the two groups (*p* = 0.0007) without significant between group differences (*p* = 0.841). 

We considered potential differences in adiposity changes based on age at baseline, sex, and ethnicity. However, the inclusion of these variables into the models did not significantly affect adiposity measures.

## 4. Discussion

This opportunistic study within a randomized controlled trial sought to investigate adiposity changes after walnut supplementation for 2 years in an independently living, predominantly healthy, elderly cohort. The increase in RBC ALA content in the walnut group is a reliable indicator that participants adhered to the intervention. We previously reported a decrease in RBC ALA in the control group at one year [22], which we speculated was the result of restricting the use of ALA-rich flax. It is possible that some control group participants may have reverted to consuming these products in the second year of the study, perhaps due to perceived benefits, hence the increase in RBC ALA. 

Overall, our data indicate that ingesting an average of nearly 300 kcal from walnuts daily for 2 years (without advice on foods to be replaced when adding walnuts to the diet) does not promote weight gain or cause significant changes in body composition. Sensitivity analyses showed that weight and adiposity trends were proportionally similar for men and women. 

A tendency towards loss of lean body mass and fat gain over time has previously been reported in studies that have longitudinally assessed spontaneous adiposity changes in healthy, weight-stable elders [23,24]. One such study on free-living elderly persons of comparable mean age followed for the same period of time as our study participants reported a 0.32 kg and 0.16 kg loss in lean body mass and a concurrent 0.4% and 0.5% increase in body fat in men and women, respectively [25]. The self-reported increase in physical activity might have been due to participants’ awareness that they were being monitored and the general tendency to over-report physical activity. Superior methods of assessing physical activity such as the use of accelerometers can help validate physical activity in future long-term nut trials in free-living individuals.

Notwithstanding the high energy density of walnuts, the lack of body weight increase might be explained by several mechanisms. We have previously reported that the energy contained in walnuts was offset in part by ≈19% spontaneous reduction in caloric intake from other food sources [22], although the compensatory response of our study subjects was lower than previously reported [26,27]. Other possible mechanisms include increased satiety following nut intake [28], energy regulation by nuts [29,30], and inefficient energy absorption from nuts [31] leading to increased fecal fat excretion [30,32,33,34]. Concerning increased fecal fat, it has been demonstrated that as much as 10–20% of the total energy from nuts is lost due to limited bioavailability in the gut [35]. In confirmation, recent findings show that the metabolizable energy content of walnuts is approximately 5.22 kcal/g (146 kcal/serving) as compared to the Atwater-calculated amount of 6.61 kcal/g (185 kcal/serving). Thus, Atwater factors overestimate by 21% the metabolizable energy content of walnuts [36]. Food compensation, increased satiety and reduced available energy are the most likely factors accounting for a stable weight during chronic nut consumption.

Our study has limitations. The original study was designed to assess changes in cognitive function and retinal health [14] and our results derive from a post hoc analysis. Also, three different clinical investigators obtained body measurements, suggesting that the data collected may be subject to interobserver variability despite the use of standardized protocols. Our study also has strengths. To the best of our knowledge, this study is the longest and largest randomized controlled trial to examine body weight change in relation to nut consumption in free-living healthy elders. Our parallel design is best suited for assessing weight changes since it disallows the potential for carry-over effects commonly seen in crossover feeding studies. Also, compliance with walnut consumption was corroborated with objective biomarkers. Future studies should consider examining whether walnuts contribute to energy regulation by increasing resting energy expenditure. Objective assessment of physical activity, i.e., using accelerometers, should assist in determining precisely the extent to which physical activity influences changes in body weight and adiposity measures in the context of chronic nut consumption.

## 5. Conclusions

In conclusion, our findings indicate that walnuts can be incorporated into the daily diet of healthy elders without concern for adverse effects on body weight or body composition. Even so, we recognize that individual differences in energy utilization and nutrient absorption and metabolism do exist, a reason why results may vary from person to person.

## Figures and Tables

**Table 1:** Baseline characteristics of participants by intervention group.

Variable		Walnut	Control	*p*-Value
*n* (%)		183 (51.4)	173 (48.6)	--
Age—year. (mean ± SD)		69.7 (4.1)	69.1 (3.7)	0.137 ^a^
Sex—no. (%)	Women	119 (65.0)	118 (68.2)	0.525 ^b^
	Men	64 (35.0)	55 (31.8)	
Ethnicity—no. (%)	White	144 (78.7)	131 (75.7)	0.221 ^a^
	Non-white	39 (21.3)	42 (24.3)	
Height—cm		167.2 (9.8)	165.9 (8.8)	0.176 ^a^
Weight—kg		77.1 (17.2)	75.6 (16.1)	0.348 ^a^
Body mass index (BMI)—kg/m^2^		27.5 (4.8)	27.4 (4.8)	0.833 ^a^
Waist circumference—cm		99.2 (14.1)	98.4 (13.4)	0.615 ^a^
Smoking—no. (%)	Never	174 (95.1)	169 (97.7)	0.503 ^c^
	Former	7 (3.8)	3 (1.7)	
	Current	2 (1.1)	1 (0.6)	
Physical activity—METs-h/week		3.54 (3.5)	3.70 (3.4)	0.840 ^a^

Data are expressed as mean (SD), except for qualitative variables, expressed as *n* (%). ^a^ Two-sample *t*-test; ^b^ Chi-square test for independence; ^c^ Fisher’s exact test.

**Table 2:** Average daily intake of macronutrients at 2 years by intervention group in participants completing the trial.

Variable	Walnut (*n* = 159)	Control (*n* = 148)	*p*-Value ^d^
	Mean * (SD)	Mean * (SD)	
Energy (kcal)	1821 (503)	1593 (423)	<0.0001
Total carbohydrate (g)	204 (76)	192 (64)	0.199
Total protein (g)	70 (18)	65 (19)	0.011
Vegetable protein (g)	30 (11)	24 (11)	<0.0001
Total fat (g)	84 (24)	63 (20)	<0.0001
Saturated fat (g)	22 (9)	21 (9)	0.185
Monounsaturated fat (g)	25 (8)	22 (7)	0.001
Polyunsaturated fat (g)	31 (8)	14 (5)	<0.0001
Dietary cholesterol (mg)	202 (102)	218 (114)	0.308
Total dietary fiber (g)	24 (10)	20 (8)	<0.0001
Total carbohydrate (% E)	42.8 (10.2)	47.3 (11.4)	<0.0001
Total protein (% E)	15.5 (5)	16.6 (5.6)	0.01
Total fat (% E)	40.2 (8.7)	33.6 (9.7)	<0.0001
Saturated fat (% E)	10 (3.9)	11 (4.9)	0.01
Monounsaturated fat (% E)	11.8 (3.7)	11.9 (4.4)	0.662
Polyunsaturated fat (% E)	15.1 (4.7)	7.8 (3.7)	<0.0001

* Mean values for five 24-h diet recalls per individual; ^d^ Two sample *t*-test for group differences; % E denotes macronutrient intake as percent of total energy.

**Table 3:** Adiposity and physical activity during the 2-year follow-up by intervention group.

Variable	Timepoint	Walnut (*n* = 159)	Control (*n* = 148)	*p*-Value ^e^	
		Mean (95% CI)	Mean (95% CI)	Time Effect	Group × Time Interaction Effect
Weight—kg	Baseline	77.1 (74.5, 79.6)	75.6 (73.0, 78.2)	0.031	0.671
	Year 1	76.9 (74.4, 79.4)	75.3 (72.7, 77.9)		
	Year 2	76.7 (74.1, 79.2)	75.0 (72.4, 77.6)		
Body fat—kg	Baseline	25.5 (24.4, 26.7)	25.5 (24.3, 26.2)	0.0001	0.528
	Year 1	25.9 (24.9, 27.0)	25.7 (24.6, 26.8)		
	Year 2	26.4 (25.3, 27.4)	26.0 (24.8, 27.1)		
Lean body mass—kg	Baseline	51.2 (49.4, 53.0)	49.5 (47.6, 51.3)	0.220	0.740
	Year 1	51.0 (49.2, 52.7)	49.4 (47.6, 51.2)		
	Year 2	50.8 (49.0, 52.6)	49.3 (47.4, 51.1)		
Waist circumference—cm	Baseline	99.4 (97.3, 101.6)	98.6 (96.4, 100.8)	0.680	0.651
	Year 1	99.6 (97.5, 101.7)	98.6 (96.5, 100.8)		
	Year 2	99.7 (97.6, 101.8)	98.6 (96.4, 100.8)		
Waist-to-hip ratio	Baseline	0.93 (0.91, 0.94)	0.92 (0.91, 0.94)	0.697	0.160
	Year 1	0.93 (0.92, 0.94)	0.92 (0.90, 0.93)		
	Year 2	0.93 (0.92, 0.95)	0.91 (0.90, 0.93)		
PA-METS—h/week	Baseline	3.54 (3.06, 4.02)	3.70 (3.21, 4.19)	<0.001	0.841
	Year 1	3.83 (3.40, 4.25)	4.02 (3.58, 4.46)		
	Year 2	4.11 (3.62, 4.61)	4.34 (3.83, 4.85)		

PA denotes physical activity; METS, metabolic equivalents. ^e^ Linear mixed models with three timepoints (baseline, year 1, and year 2). Model includes time, intervention, and their interaction. Results are adjusted for in-trial changes in PA.
